# Allegation Assessment and Outcome Differences Across Racial Groups From Standardized Adult Protective Services Data

**DOI:** 10.1093/geroni/igaa057.2322

**Published:** 2020-12-16

**Authors:** Zachary Hass, Pi-Ju (Marian) Liu, Karen Conrad, Sara Stratton, Kendon Conrad

**Affiliations:** 1 Purdue University, West Lafayette, Indiana, United States; 2 University of Illinos at Chicago, Chicago, Illinois, United States; 3 San Francisco Adult Protective Services, San Francisco, California, United States; 4 University of Illinois at Chicago, Chicago, Illinois, United States

## Abstract

Objective assessment is an important tool for Adult Protective Services (APS) in supporting a diverse population. Out of the 1472 APS’ clients, aged 65 and over, assisted during the study period, 39% identified as a non-white race and 30% did not speak English. Providing services to this vulnerable population is made even more difficult by the need to provide culturally appropriate services. In this work, we present on differences in types of abuse alleged, abuse severity assessment, services provided, and preliminary outcomes across racial and language groups. For example, for this population, clients identifying as black race had the highest allegation rates of neglect (16%) and financial abuse (27%), Asians of physical (15%) and emotional abuse (24%), and other races of self-neglect (56%) and isolation (9%). Standardized data collection permits tracking such patterns and objective assessment tools help to avoid systemic bias in aiding this vulnerable population.

